# Emotional dissonance and mental health among home-care workers: A nationwide prospective study of the moderating role of leadership behaviors

**DOI:** 10.5271/sjweh.4197

**Published:** 2025-01-01

**Authors:** Håkon A Johannessen, Morten Birkeland Nielsen, Rigmor Harang Knutsen, Øivind Skare, Jan Olav Christensen

**Affiliations:** 1 Research Group for Work Psychology and Physiology, National Institute of Occupational Health in Norway, Oslo, Norway.; 2 Department of Psychology, University of Oslo, Oslo, Norway.; 3 Research Group for Occupational Medicine and Epidemiology, National Institute of Occupational Health in Norway, Oslo, Norway.

**Keywords:** common mental disorder, emotion work, emotional demand, job demand, leadership, prospective, stress, well-being, work

## Abstract

**Objectives:**

Evidence suggests that emotional dissonance, the imbalance between true feelings and those displayed to meet work standards, heightens the risk of mental distress. In nursing occupations, exerting such emotional effort is a part of the job role. Drawing from the job demands–resources model, high-quality leadership is a resource that may assist employees in coping with stressors. We examined whether quality of leadership mitigated the potential adverse impact of emotional dissonance on mental health.

**Methods:**

In 2019, 1426 home-care workers from 130 organizational units were surveyed, with follow-ups after 8 and 14 months. Prospective associations between emotional dissonance (the Frankfurt Emotion Work Scales) and mental distress (Hopkins Symptom Checklist, HSCL-5), including interactions between emotional dissonance and leadership behaviors (Nordic Questionnaire for Psychological and Social Factors at Work), were determined using lagged linear mixed models.

**Results:**

Emotional dissonance was positively associated with mental distress (adjusted P<0.05), whereas supportive, empowering, and fair leadership were negatively associated with mental distress (adjusted P<0.05). All three investigated sources of leadership behaviors moderated the direct association between emotional dissonance and mental distress (adjusted P<0.05). Emotional dissonance and mental distress were reciprocally related; an increase in either will heighten the level of the other. Leadership behaviors did not moderate the reversed association between emotional dissonance and mental distress (adjusted P>0.05).

**Conclusions:**

Supportive, empowering, and fair leadership buffers the association of emotional dissonance on mental distress. Strategic interventions that enhance the quality of leadership may help prevent mental distress among employees in professions with emotionally demanding tasks.

Mental health problems and disorders are widespread in the working population, emerging as a leading cause of sickness absence and permanent withdrawal from the workforce in many high-income countries (1–3). These conditions carry significant implications, ranging from diminished quality of life for affected individuals to reduced productivity and organizational performance for employers, along with increased expenditures for the welfare state (2). The majority of mental health issues identified among the working population are common mental disorders such as depression, anxiety, and substance-use disorders, which may be preventable (4). Exposure to adverse factors at work has been linked to an elevated risk of common mental disorders (4), and the level of these factors, as well as the prevalence of common mental disorders, varies between occupations (5, 6). Within the nursing workforce, common mental disorders are particularly prevalent (5, 7, 8). Gaining insight into the mechanisms linking specific characteristics of nursing work to mental health can facilitate the development of preventive measures against adverse health effects of work factors for an essential occupational group.

In nursing work like home-care, particular characteristics of the work are emotionally demanding, such as serving the needs and dealing with the problems and suffering of clients and patients (9, 10). Moreover, in the course of their professional duties, nursing and home-care staff are expected to regulate their emotions and express appropriate emotions as part of their job requirement, irrespective of their genuine emotional state. This includes expressing empathy even when they might be fatigued or dealing with challenging or difficult patients (11). The resulting mismatch between felt emotions and the emotions expressed to conform to professional standards, social norms, and organizational requirements is referred to as emotional dissonance (11, 12). Hochschild (13) originally introduced key aspects of this construct when she labelled the phenomena emotional labor and hypothesized that requirements of showing emotions not felt in the situation would lead to the alienation of one's feelings, which in turn would cause mental health problems such as mental distress. Increasing epidemiologic evidence show that emotionally demanding work is linked to excess risk of developing mental distress (14, 15).

In home-care service work, emotionally demanding tasks stem from characteristics of the work that may not be amenable to change, given that an essential or inherent part of the job is to deal with clients and patients who often are in a difficult or vulnerable situation (9). Hence, to prevent employee ill-health in home-care work, it is crucial to examine the existence of modifiable or promotable work environmental factors that can buffer potential detrimental effects of emotional dissonance on mental health. Leadership, defined as the process whereby an individual influences a group of individuals to achieve a common goal (16), is one factor that is considered amenable to strategic interventions and frequently studied as a factor potentially affecting employees’ health (17, 18). According to the job demands–resources model, high quality leadership is a vital resource in the work environment that can assist employees in coping with stressors (19). Specifically, high quality leadership behaviors are likely to provide the needed empathy, compassion, support, and guidance that influence employees’ well-being in response to emotional demands. Such behaviors can also inspire employees to overcome psychological setbacks and instill in them the strength to tackle future hurdles (20).

Hence, it is reasonable to propose that leadership quality buffers the detrimental effects of emotional dissonance on employee mental health. However, there is a lack of studies that have addressed whether aspects of leadership mitigate the elevated risk of mental health outcomes due to high emotional demands or dissonance. Upon reviewing the literature, we identified only one prospective study in the last 15 years (9).

In the literature examining how leadership impacts employees’ health and well-being, two main approaches emerge: one emphasizing task-oriented aspects of leadership and the other focusing on relation-oriented dimensions of leadership (16). To achieve the common goal, task-oriented leaders focus on the task to be accomplished by the followers, whereas relation-oriented leaders focus on the quality of the relationship with followers. In a systematic review of nursing studies, the authors concluded that leadership focused on task completion alone is not sufficient to achieve optimal outcomes for the nursing workforce, but a focus on relational leadership is needed to achieve healthy work environments (16).

The current study aimed to determine whether aspects of relation-oriented leadership, namely fair-, empowering-, and supportive-leadership behaviors, moderated prospective associations of emotional dissonance on employee mental health. Fair leadership embodies a steadfast commitment to upholding procedural justice and ethical standards, ensuring transparency in decision-making and equitable treatment (21). Empowering leaders typically prioritize fostering employee participation, skill enhancement, and enabling individuals (21). Supportive leaders distinguish themselves by their attentive and considerate approach towards their employees (21). These three dimensions are considered separate, but related aspects of relation-oriented leadership behaviors (22).

There are good reasons to expect that these aspects of leadership behaviors may contribute to increasing employees’ abilities to cope with high emotional demands. Fair leadership may increase trust in the supervisor and can foster healthy self-worth among employees (23). Moreover, meta-analytic evidence shows that fairness relates to positive affective states among employees (24). Empowerment may enhance subordinates’ decision authority, and sense of self-efficacy by providing them with the resources needed to cope effectively (25), while supportive leaders can provide emotional encouragement along with coping assistance in terms of information, advice, or active coping strategies such as threat re-appraisal (26).

Based on the above, three main hypotheses were posed: There is a prospective positive association between emotional dissonance and mental distress (H1); there are prospective negative associations between fair, empowering, and supportive leadership and mental distress (H2); and fair, empowering, and supportive leadership moderate the prospective positive association between emotional dissonance and mental distress (H3).

## Methods

### Design and study sample

This is an observational study utilizing data from an intervention project aimed at determining the effects of regulatory tools on working conditions. The aims and design of the intervention project are detailed in the study protocol (27). The intervention project did not find any notable differences between the intervention and control groups regarding psychosocial work factors or subjective health outcomes (28, 29).

Participants were surveyed for psychosocial work factors and subjective health outcomes at baseline, with follow-ups conducted at 8 and 14 months. Clusters in the study were municipalities, Norway’s base administrative units that are responsible for providing primary healthcare, including home-care. To reduce intra-cluster variability, eligible municipalities employed 20–100 home-care workers (27). As of January 2019, of Norway’s 422 municipalities, 187 met the eligibility criteria. Based on power calculations, 132 were randomly selected and assigned to one of four trial arms and then informed about the study by letter and email. By May 2019, 96 municipalities had agreed to participate, and invitations were extended to all public home-care workers within these municipalities, including both home-care nurses (providing professional medical care) and home-care aides (assisting with personal care and housekeeping). To increase statistical power due to a low response rate, a decision was made to expand the eligibility criteria for municipalities to include those employing 101–200 home-care workers. As a result, an additional 48 municipalities were recruited in June 2019, of which 34 agreed to participate. This extension resulted in a total of 130 participating municipalities out of 180 invited (72%), with 2591 of 7103 (36%) invited home-care workers consenting to participate at baseline. Of these, 1426 took part in this study at baseline and at 8 and/or 14 months follow up, ie, 2 months before the intervention, and 6 and 12 months after the intervention. Data were collected from each individual participant using a proprietary web-based questionnaire comprising established psychometric instruments to measure the variables of interest. The questionnaire could be completed in multiple sessions and accessed with a unique code distributed to each participant in advance.

### Variables

*Predictor: emotional dissonance.* Emotional dissonance was measured using four items from the Frankfurt Emotion Work Scales (30) (example item: “How often in your job do you have to suppress emotions in order to appear neutral on the outside?”). Responses on all items were given on a 5-point Likert scale, ranging from 1=seldom or never, 2=once per week, 3=once per day, 4=several times per day, and 5=several times an hour. Cronbach’s α was 0.86 at t1, 0.87 at t2, and 0.89 at t3. Zapf et al (12) has shown evidence for criterion-related validity of the scale.

*Predictor and moderator: leadership behaviors.* The three dimensions of leadership behaviors (fairness, empowerment, and support) were assessed by validated scales from the General Nordic Questionnaire for Psychological and Social Factors at Work (QPS_Nordic_) (21). Responses on all items were given on a 5-point Likert scale, ranging from 1=very seldom or never to 5=very often or always. Cronbach’s α values were as follows: support from the immediate superior (three items; t1 α=0.89, t2 α=0.88, t3 α=0.91), empowering leadership (three items; t1 α=0.88, t2 α=0.88, t3 α=0.89), and fair leadership (three items; t1 α=0.78, t2 α=0.81, t3 α=0.78)

### Covariates

Age, sex (male or female), years of education, and percentage of full-time equivalent position were included as potential confounders.

### Outcome: mental distress

Depression and anxiety symptoms were assessed using a 5-item version of the Hopkins Symptoms Checklist (HSCL-5). The abbreviated 5-item version has recently undergone validation, demonstrating a robust correlation with the original scale (HSCL-25) and consistent performance across various demographic groups (31). Participants were asked to rate the following symptoms of depression and anxiety in the past 14 days on a 4-point scale: (i) feeling fearful; (ii) nervousness or shakiness inside; (iii) feeling hopeless about the future; (iv) feeling blue; and (v) worrying too much about things. The four response categories were: ‘Not been affected at all’=1, ‘Not been affected much’=2, ‘Been affected quite a lot’=3, and ‘Been affected a great deal’ =4. Higher scores indicate higher levels of psychological distress. The computed index represented the mean of these item scores. Cronbach’s α was 0.88 at t1, 0.88 at t2, and 0.88 at t3. For descriptive purposes only, cases of mental distress were identified by a cut-off point of ≥2.0 on the HSCL-5, as suggested by a previous study comparing its performance to the 25-item version (32).

### Statistical analyses

All analyses were conducted using STATA (version 16.1, Stata Corp, College Station, TX, USA), and significance was set at P<0.05. To determine whether emotional dissonance impacts mental distress and assess the potential moderating role of leadership behaviors, we employed lagged linear mixed models. All variables were assessed at baseline (t1), and at 8 (t2) and 14 (t3) months follow-up. Emotional dissonance and leadership behaviors, including their interaction terms, were entered into the model at time t–1 (t1 and t2), whereas mental distress was entered at time t (t2 and t3). The mixed models incorporated emotional dissonance, leadership behaviors, and their interaction terms as fixed effects. Random intercepts were used to correct for the clustering of variables in subjects. Both crude models and models adjusted for age, sex (male or female), years of education, and percentage of full-time equivalent position were computed. These covariates were selected a priori based on their potential influence on the associations of interest. Initially, separate models were fitted for each work-related predictor. Subsequently, interaction effects between emotional dissonance and leadership behaviors were analyzed individually for each of the three leadership variables.

Health can impact employees’ perception of their psychosocial work environment and/or their ability to cope with job demands (33, 34). Hence, stressor-strain relations, as well as reversed and reciprocal relations, are likely. To account for the possibility of reverse associations, we computed the following model, in which mental distress and leadership behaviors were entered as predictors at time t–1 (t1 and t2), while emotional dissonance was treated as an outcome at time t (t2 and t3).

Interaction effects were plotted by mean centering the predictor variables and generating predicted means of the dependent variable for each combination of one standard deviation below the mean, one standard deviation above the mean, and at the mean on the centered predictors.

To address potential selection bias arising from participant dropouts between the baseline assessment and follow-ups, we conducted t-tests for continuous variables and chi squared tests for binary variables to examine differences between background characteristics, exposure levels, and outcomes at baseline between responders at follow-up and those who dropped out. To determine if the association’s robustness might be affected by the selection of participants who dropped out, we assessed the strength of bivariate cross-sectional associations between the predictors and mental distress at baseline in the two groups.

## Results

Sample characteristics of respondents from the first (baseline), second, and third surveys are shown in table 1. The vast majority of respondents at baseline were female (95%), and the mean age was 45 years. The level of education was centered on upper secondary education (49%) and university or college (44%) education. In total, 79% of the respondents were in full-time employment, and 15% reported clinically relevant symptoms of mental distress (HSCL ≥2).

**Table 1:** Sample characteristics at baseline, 8- and 14-months follow-up. [SD=standard deviation; HSCL=Hopkins Symptom Checklist]

		Respondents at t1 (N=2591)					Respondents at t2 (N=1319)					Respondents at t3 (N=694)			
		N	%	Mean	SD		N	%	Mean	SD		N	%	Mean	SD
Sex															
	Male	128	4,9				59	4.5				32	4.6		
	Female	2463	95.1				1260	95.5				662	95.4		
Age				44.6	12.1				45.5	11.6				46.1	11.1
Education (years)															
	1–9	84	3.4				41	3.2				17	2.6		
	10–12	1214	48.9				574	45.3				310	46.8		
	13–16	1079	43.5				592	46.7				309	46.7		
	>16	106	4.3				60	4.7				26	3.9		
	Missing	108					52					32			
Percentage of full-time equivalent employment				79.4	22.5				82.7	20.3				82.1	20.1
Emotional dissonance (range 1–5)				2.37	0.94				2.42	0.95				2.45	0.98
Supportive leadership (range 1–5)				3.76	1.04				3.71	1.04				3.67	1.07
Empowering leadership (range 1–5)				3.19	1.06				3.17	1.04				3.07	1.05
Fair leadership (range1–5)				4.00	0.86				3.95	0.88				3.91	0.89
Mental distress (range 1–4)				1.41	0.55				1.44	0.56				1.41	0.54
	HSCL ≥2	331	15.2				169	16.5				89	15.9		
	Missing	409					292					135			

Compared to those who responded at follow up, respondents who dropped out after the baseline measurements were largely similar in demographic and work characteristics (table 2). There were no differences in terms of sex, education, or the mean score on emotional dissonance, leadership behaviors, and mental distress. However, respondents who dropped out were slightly younger and had a slightly lower percentage of full-time equivalent employment. The magnitudes of the crude cross-sectional associations between emotional dissonance and mental distress were similar and statistically significant for both those who responded at follow-up and those who dropped out (table 3). Although the crude cross-sectional associations between the leadership variables and mental distress were statistically significant in both groups, the magnitude of these associations was stronger among those who responded at follow up (table 3).

**Table 2:** Sample differences between follow-ups and dropouts, measured at baseline. [SD=standard deviation.]

		Respondents at follow up (N=1426) ^a^					Dropouts at follow up (N=1113) ^b^					Sample differences	
		N	%	Mean	SD		N	%	Mean	SD		t-test (P-value)	X^2^ (P-value)
Sex													>0.05
	Male	64	4.5				61	5.5					
	Female	1362	95.5				1052	94.5					
Age				45.4	11.6				43.4	12.5		<0.05	
Education (years)													>0.05
	1–9	39	2.9				43	4.0					
	10–12	645	47.5				545	50.7					
	13–16	616	45.4				442	41.1					
	>16	58	4.3				45	4.2					
	Missing	68					38						
Percentage of full-time equivalent employment				81.3	20.9				76.7	24.3		<0.05	
Emotional dissonance (range 1–5)				2.39	0.94				2.37	0.94		>0.05	
Supportive leadership (range 1–5)				3.79	1.03				3.73	1.03		>0.05	
Empowering leadership (range 1–5)				3.22	1.05				3.15	1.07		>0.05	
Fair leadership (range 1–5)				4.00	0.85				3.97	0.89		>0.05	
Mental distress (range 1–4)				1.39	0.53				1.43	0.57		>0.05	
	HSCL ≥2	183	14.2				136	16.0				>0.05	
	Missing	140					261						

^a^ Respondents at baseline and at 8 months, 14 months, or both. ^b^ Respondents at baseline only.

**Table 3:** Crude associations between exposure variables and mental distress at baseline. [CI=confidence interval.]

	Responders at follow up			Dropouts at follow up	
	Coefficients	95% CI		Coefficients	95% CI
Emotional dissonance	0.19	0.16–0.22		0.18	0.14–0.22
Supportive leadership	-0.17	-0.19– -0.14		-0.09	-0.13– -0.06
Empowering leadership	-0.14	-0.17–-0.11		-0.09	-0.12– -0.05
Fair leadership	-0.21	-0.24– -0.18		-0.16	-0.20– -0.11

**Figure 1–3:**
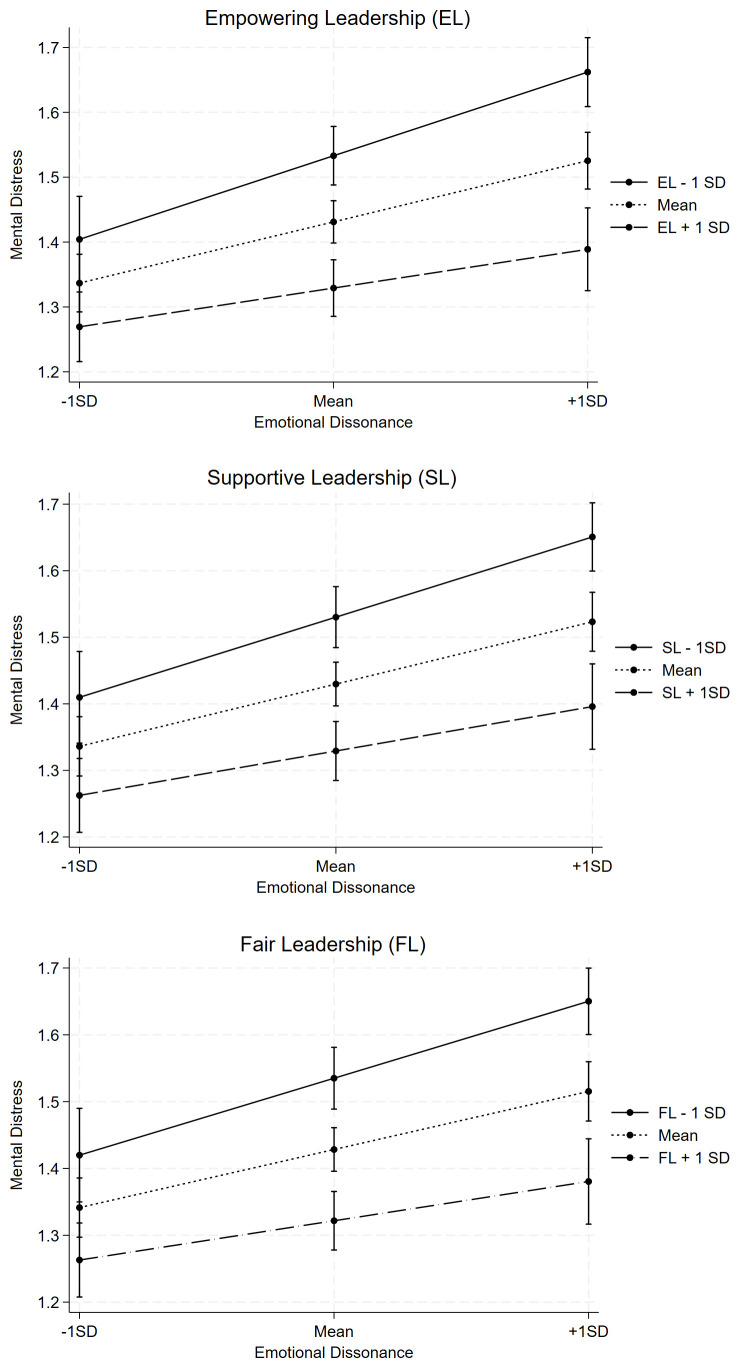
Predicted means of mental distress for different values of leadership behaviors and emotional demands. [SD=standard deviation; EL=empowering leadership; FL=fair leadership; SL=supportive leadership.]

In the prospective analyses, emotional dissonance was positively associated with mental distress (H1), whereas supportive, empowering, and fair leadership behaviors were negatively associated with mental distress (H2) (table 4; adjusted P<0.05). Figures 1–3 show that the three examined indicators of leadership behaviors moderated the prospective association between emotional dissonance and mental distress. The interaction coefficients were statistically significant for all three leadership variables (H3) (table 4, adjusted P<0.05).

**Table 4:** Effects of emotional dissonance (ED) and leadership behaviors on mental distress. [SE=standard error]

		Crude models				Adjusted models ^a^		
		Coefficients	SE	P-value		Coefficients	SE	P-value
Main effects								
	Emotional dissonance	0.125	0.015	0.000		0.122	0.016	0.000
	Supportive leadership (SL)	-0.118	0.014	0.000		-0.120	0.015	0.000
	Empowering leadership (EL)	-0.112	0.014	0.000		-0.116	0.015	0.000
	Fair leadership (FL)	-0.149	0.017	0.000		-0.154	0.017	0.000
Interaction effects								
	Emotional dissonance	0.205	0.052	0.000		0.099	0.016	0.000
	Supportive leadership	-0.030	0.037	0.428		-0.097	0.015	0.000
	ED×SL	-0.027	0.013	0.044		-0.028	0.014	0.049
	Emotional dissonance	0.223	0.044	0.000		0.100	0.016	0.000
	Empowering leadership	-0.022	0.035	0.912		-0.097	0.015	0.000
	ED×EL	-0.038	0.013	0.004		-0.035	0.014	0.010
	Emotional dissonance	0.256	0.063	0.000		0.092	0.016	0.000
	Fair leadership	-0.022	0.044	0.628		-0.123	0.018	0.000
	ED×FL	-0.040	0.016	0.010		-0.034	0.016	0.036

^a^ Adjusted for sex, age, education, and percentage employment.

Table 5 shows that there was a reversed positive association between emotional dissonance and mental distress (adjusted P<0.05). Moreover, the three indicators of leadership behaviors were, in prospective analyses, negatively associated with emotional dissonance (adjusted P<0.05). The three indicators of leadership behaviors did not moderate the reversed association between emotional dissonance and mental distress (table 5, adjusted P>0.05).

**Table 5:** Reverse associations: mental distress (MD) and leadership behaviors as predictors of subsequent emotional dissonance. [SE=standard error]

		Crude models				Adjusted models ^a^		
		Coefficients	SE	P-value		Coefficients	SE	P-value
Main effects								
	Mental distress	0.386	0.043	0.000		0.359	0.045	0.000
	Supportive leadership (SL)	-0.154	0.066	0.000		-0.159	0.024	0.000
	Empowering leadership (EL)	-0.117	0.023	0.000		-0.126	0.024	0.000
	Fair leadership (FL)	-0.199	0.028	0.000		-0.203	0.028	0.000
Interaction effects								
	Mental distress	0.220	0.128	0.084		0.211	0.130	0.104
	Supportive leadership	-0.167	0.058	0.004		-0.167	0.059	0.005
	MD×SL	0.035	0.035	0.317		0.028	0.036	0.430
	Mental distress	0.324	0.120	0.007		0.321	0.123	0.009
	Empowering leadership	-0.096	0.061	0.114		-0.094	0.062	0.130
	MD×EL	0.011	0.040	0.789		-0.001	0.041	0.975
	Mental distress	0.154	0.149	0.302		0.131	0.152	0.389
	Fair leadership	-0.221	0.067	0.001		-0.225	0.068	0.001
	MD×FL	0.049	0.040	0.220		0.046	0.040	0.253

^a^ Adjusted for sex, age, education, and percentage employment

## Discussion

Based on a large prospective nationwide sample of home-care workers in Norway, we aimed to determine if high-quality leadership mitigated the potential adverse impact of emotional dissonance on mental health. In line with a growing number of prospective studies, we found that emotional dissonance was positively associated with mental distress (14, 15), whereas high-quality leadership behaviors were negatively associated with mental distress (18, 35). The present study adds to the research literature by providing evidence for a buffering effect of high-quality leadership behaviors on the relationship between emotional dissonance and mental distress.

Our findings align with the job demands–resources theory, which posits that job demands and resources activate distinct processes (19). Job demands may trigger stress or health impairment, while job resources initiate a motivational process linked to positive outcomes such as goal achievement, personal growth, and development. Following the job demands–resources model, if high demands persist without adequate compensation from job resources, employees’ energy is gradually depleted, potentially leading to mental exhaustion and distress (36). High leadership quality, serving as a significant job resource or enhancer of other resources, such as control, role clarity, and intrapersonal motivation, holds the potential to mitigate the adverse effects of elevated emotional demands on employees’ mental health.

Our analyses revealed a reciprocal relationship between emotional dissonance and mental distress, meaning each can predict the other over time. In other words, the onset of either increased emotional dissonance or mental distress appears to heighten the level of the other. One might speculate that increased emotional dissonance gradually depletes employees’ energy, leading to exhaustion and distress. This exhaustion, in turn, makes it harder to mobilize the resources needed to manage emotionally demanding work, thereby shifting both their perception and experience of these demands. The result is a vicious cycle, in which emotional dissonance and mental distress exacerbate each other (33, 34).

Although the stressor-strain association is commonly discussed, reverse and reciprocal relationships are well-documented in the literature on psychosocial factors and health (33, 34). This is based on the accepted assumption that individuals actively shape their environment through perceptual and/or behavioral mechanisms, rather than being passively influenced by it (34).

Our results also indicate that supportive, empowering, and fair leadership behaviors were negatively associated with emotional dissonance, but these behaviors did not moderate the relationship between mental distress and emotional dissonance. Hence, leadership behaviors do not seem to alter the perception or experience that employees with increased levels of mental distress tend to have—namely, viewing or experiencing emotionally demanding work tasks as more strenuous. This highlights the importance of prioritizing primary preventive strategies to address work environment challenges related to emotionally demanding tasks and mental health.

To the best of our knowledge, this is the first prospective study to provide empirical evidence on the buffering role of leadership behaviors on the prospective association between emotional dissonance at work and mental health. Previous prospective studies have examined whether leadership quality buffer the effects of emotional demands on the risk of antidepressant treatment and long-term sick leave, respectively. Madsen et al (9) showed that leadership quality did not mitigate the association between emotional demands and antidepressant treatment. However, that study was limited by the use of a non-validated scale of leadership quality, possibly explaining the lack of both main and buffering effects. Rugulies et al (37) found no evidence that high leadership quality buffered the association between emotionally demanding work and the risk of long-term sick leave, with a possible explanation for the null finding pertaining to the lack of information on the diagnoses causing the sick leave.

The current study affirms the high prevalence of clinically relevant symptoms of anxiety and depression among home-care workers. Within our baseline sample, 15% experienced such symptoms, which is six percentage points higher when compared to the general working population in Norway (38). Furthermore, data from Statistics Norway show that employees in the home-care sector in Norway report the highest exposure to emotional demands at work (38). The robust prospective association between the experience of emotionally demanding job tasks and mental distress in the present study may contribute to explaining the high levels of distress among workers in this sector.

These job demands are inherent to the nature of the work performed by home-care workers and are not easily modifiable, originating from specific aspects of job tasks, such as addressing the challenges and distress of patients and clients. Therefore, our finding regarding the buffering effect of high-quality leadership behaviors is noteworthy, as it proposes a viable preventive strategy involving the prioritization of initiatives to improve leadership standards. High-quality leadership may directly contribute to improving mental health as well as alleviating the negative mental health impacts of emotional dissonance. This holds significant importance, especially in light of prior research on home-care workers, which indicates that approximately 30% of sick leave cases due to common mental disorders can be attributed to high levels of emotionally demanding job tasks (7).

Prior research shows that a 10% reduction in the average HSCL-5 scale score roughly corresponds to a six percentage point decrease in the number of individuals with clinically significant symptoms of depression and anxiety (HSCL-5 score ≥ 2) (27). Our findings demonstrate that for individuals with emotional dissonance scores one standard deviation above the mean, the HSCL-5 score varied by approximately 15%, depending on whether their scores were one standard deviation above or below the mean on leadership variables, as depicted in figures 1–3. Hence, we can outline that the protective effect of high-quality leadership behaviors on the association between emotional dissonance and clinically relevant symptoms of depression and anxiety (HSCL-5 score ≥ 2) is also notable.

There is evidence that various aspects of leadership can be improved by targeted interventions (39) (17, 40). A 2023 umbrella review identified several systematic reviews providing evidence that different interventions aimed at increasing leadership quality (eg, changes in management approach or management development) are successful (17). However, subsequent evidence regarding improved leader quality on the work environment and employee health outcomes showed varying effects, as most studies did not measure worker outcomes. Regarding healthcare employees, a 2021 review concluded that the most promising strategy to promote workers mental health was interventions aimed at enhancing various aspects of leadership competencies (41).

In general, there is a shortage of prospective studies examining whether leadership moderates the well-established connection between psychosocial stressors at work and general health outcomes (42), as well as emotional dissonance and mental distress specifically (9). Despite this, evidence supports the notion that fair, empowering, and supportive leadership is linked to a decreased risk of ill health and mental distress (35, 43–45). Furthermore, considering the evidence that healthier employees who perform work tasks more efficiently (46) are less prone to job turnover (47), exhibit a higher likelihood of engaging in extra-role behaviors (48), and express greater job satisfaction (49), there is a compelling rationale for home-care organizations to prioritize efforts aimed at enhancing leadership competencies.

### Strengths and limitations

This study utilized a large nationwide prospective sample with three measurement points and employed valid, well-established instruments, ensuring high reliability and internal validity. We applied mixed models, which are well-known and widely used for analyzing longitudinal data (50). A key advantage of these models is their ability to control for unobserved time-invariant variables that could confound relationships (51). While adjusting for lagged values of the dependent variable is common practice when examining causal processes within occupational health psychology (52), we chose not to apply this approach in our models. Both conceptual (53) and statistical arguments (52) raise concerns about the validity of this practice in observational studies. In the context of our study, it is likely that pre-existing levels of emotional dissonance influenced baseline levels of mental distress. Consequently, baseline mental distress may act as a mediator, and adjusting for it could lead to biased results (52–55). In mixed models, the random intercept represents the effect of all unobserved variables on the outcome and is assumed to be independent of the other predictor variables. However, if lagged values of the dependent variable are included as a predictor, the random intercept will directly affect this variable because the model applies to all time points (56). If the random intercept affects the predictor variable that comprises lagged values of the dependent variable, it cannot simultaneously be independent of it. Studies have shown that violating this assumption of independence leads to biased results (51, 54). Instead, we accounted for reverse causality by analyzing mental distress and leadership behaviors as predictors at time t–1 (t1 and t2), while treating emotional dissonance as an outcome at time t (t2 and t3).

The relatively low response rate poses a potential threat to external validity, raising concerns about the generalizability of the results to the broader population of home-care workers. Self-selection bias, arising when those who opt to respond differ systematically from non-respondents, may impact external validity. It is noteworthy that selective response can sway results, even with high response rates, whereas the validity remains unaffected by high non-response if it is random (57). Unfortunately, we lacked information to investigate whether non-responders differed systematically from responders at baseline. Moreover, attrition from t1 to t3 was considerable, possibly influenced by the known high turnover rate in the home-care sector. Unfortunately, we lacked information to investigate whether t1 responders who dropped out were still employed in the same organization at t2 and t3. Nevertheless, attrition analyses indicated no significant differences between those who responded at follow-up and those who dropped out with respect to emotional dissonance, leadership behaviors, and mental distress. Furthermore, the strength of the examined associations remained consistent in both groups.

All data were collected through self-report questionnaires due to the study’s focus on workers’ perceptions of working conditions. Given the subjective nature of emotional dissonance, the appraisal of leadership quality, and mental distress, objective assessment methods pose challenges. To address potential issues arising from self-report, such as subjective interpretations, response set tendencies, and common method variance (58), steps were taken, including varying response anchors, separating independent and dependent variables in the survey, and ensuring participant anonymity (59). Given that this study stands as a singular contribution to documenting how high quality leadership behaviors buffers the impact of emotional dissonance on mental distress, replication in diverse populations is warranted for a more comprehensive understanding. To determine if the results of this study are replicable for mental health outcomes beyond self-reporting, future research should incorporate medically determined diagnoses for anxiety and depression.

### Concluding remarks

Our nationwide study on home-care workers highlights the role of high-quality leadership in mitigating the negative impact of emotional dissonance on mental health. The study results underscore the benefits for organizations, particularly in human services where the prevalence of emotionally demanding job tasks is known to be high, of prioritizing the enhancement of leadership behaviors as a viable measure to prevent employee mental distress. Future studies should investigate whether our findings can be replicated in different occupational settings and whether they hold relevance to clinically diagnosed anxiety and depression outcomes.
